# Sucrose phosphate synthase and sucrose phosphate phosphatase interact *in planta* and promote plant growth and biomass accumulation

**DOI:** 10.1093/jxb/erv101

**Published:** 2015-04-13

**Authors:** Victoria J. Maloney, Ji-Young Park, Faride Unda, Shawn D. Mansfield

**Affiliations:** Department of Wood Science, University of British Columbia, 2424 Main Mall, Vancouver, BC, V6T 1Z4, Canada

**Keywords:** BiFC, biomass, BRET, cellulose, poplar, protein–protein interaction, sucrose phosphate phosphatase, sucrose phosphate synthase, yeast two-hybrid.

## Abstract

Bioinformatic analysis indicates that sucrose phosphate synthase (SPS) contains a putative C-terminal sucrose phosphate phosphatase (SPP)-like domain that may facilitates the binding of SPP. If an SPS–SPP enzyme complex exists, it may provide sucrose biosynthesis with an additional level of regulation, forming a direct metabolic channel for sucrose-6-phosphate between these two enzymes. Herein, the formation of an enzyme complex between SPS and SPP was examined, and the results from yeast two-hybrid experiments suggest that there is indeed an association between these proteins. In addition, *in planta* bioluminescence resonance energy transfer (BRET) was observed in *Arabidopsis* seedlings, providing physical evidence for a protein interaction in live cells and in real time. Finally, bimolecular fluorescence complementation (BiFC) was employed in an attempt to detect SPS–SPP interactions visually. The findings clearly demonstrated that SPS interacts with SPP and that this interaction impacts soluble carbohydrate pools and affects carbon partitioning to starch. Moreover, a fusion construct between the two genes promotes plant growth in both transgenic *Arabidopsis* and hybrid poplar.

## Highlight

SPS and SPP interact to form a metabolic channel that impacts sucrose metabolism and manifests in enhanced plant growth.

## Introduction

Sucrose, the major product of photosynthesis, can be utilized directly by glycolysis or translocated within the plant as a soluble carbohydrate via the phloem. It constitutes the most abundant form of soluble storage carbohydrate and also serves as a signalling molecule that triggers essential metabolic events (Koch, 1996). When imported into sink tissues, sucrose can be used for the maintenance of cellular metabolism, cell wall biosynthesis, respiration, or converted into starch for later use (Sturm, 1999; Kutschera and Heiderich, 2002). The synthesis of sucrose has been well characterized in plants and microbes, and involves a two-step process catalysed by sucrose-6-phosphate synthase (SPS; EC 2.4.1.14) and sucrose-6-phosphate phosphatase (SPP; EC 3.1.3.24). The reaction catalysed by SPS, the synthesis of sucrose-6-phosphate (Suc-6-P) from fructose-6-phosphate (Fru-6-P) and UDP-glucose, is an important regulatory step in controlling sucrose synthesis in plants (Stitt *et al.*, 1988). SPS plays a crucial role in carbohydrate metabolism by regulating the partitioning of carbon between starch production and carbohydrate accumulation in many physiological and developmental processes (Geigenberger *et al.*, 1999; Baxter *et al.*, 2003; Lunn and MacRae, 2003; Chen *et al.*, 2005). SPP catalyses the final step in the pathway of sucrose biosynthesis, where Suc-6-P produced by SPS is irreversibly hydrolysed to sucrose (Lunn and Rees, 1990). Several reports have shown that plants have multiple isoforms of SPS and SPP, and that their expression varies with development, tissue type, and environmental signals (Reimholz *et al.*, 1997; Lunn, 2003; Lutfiyya *et al.*, 2007). Protein phosphorylation of multiple serine residues and direct control via metabolic effectors have been suggested as the main regulatory mechanisms of SPS activity (Huber and Huber, 1992, 1996). In tobacco (*Nicotiana tabacum* L), two 14-3-3 protein isoforms were found to interact with SPS in a two-hybrid screen. Additionally, deletion analysis suggested that differences in protein affinity for SPS are mediated by the variable C-terminus of 14-3-3s (Bornke, 2005). While uncertainty surrounds the exact function of these proteins, it is believed that they are involved in the regulation of protein turnover (Cotelle *et al.*, 2000). It has also been proposed that additional regulation of the sucrose synthesis pathway could be facilitated by the formation of a multienzyme complex between SPS and SPP. This was first suggested by Salerno *et al.* (1996) who identified a protein that altered the catalytic activity of SPS, and the protein was tentatively identified as SPP via co-migration through gel filtration and anion-exchange chromatography. Moreover, Echeverria *et al.* (1997) who, by measuring the rates of Suc-6-P synthesis and hydrolysis in mixing experiments with partially purified enzymes from spinach (*Spinacia oleracea*) and rice (*Oryza sativa*) leaves, provided kinetic evidence for their association. Altered migration of the two proteins on non-denaturing gels and an indication of metabolite channelling of Suc-6-P provided additional evidence for a close association between SPS and SPP.

In addition to physical and kinetic evidence, phylogenetic analysis of both enzymes has revealed that SPS contains an SPP-like domain at the C-terminus to which SPP may bind. It has been suggested that a gene fusion of a glucosyl-transferase-like domain (GTD) and a phosphohydrolase-like domain (PHD) might have given rise to a hypothetical common ancestral *SPS* gene, while duplications of PHD and GTD during cyanobacterial diversification might have produced *SPP* and sucrose synthase (*SUSY*) genes, respectively (Cumino *et al.*, 2002; Lunn and MacRae, 2003). Aravind *et al.* (1998) reported that the N-terminal region of the maize SPP1 shows significant similarity to proteins in the haloacid dehalogenase (HAD) superfamily of phosphatases/hydrolase. The splitting of SPS and SPP into two different polypeptides could have provided a new level of regulation that allowed protein–protein interactions and the channelling of the intermediate product (Suc-6-P) (Salerno and Curatti, 2003). It has also been shown that chimeric *Synechocystis* SPS–SPP protein expressed in a heterologous *Escherichia coli* system displayed SPS and SPP activity. However, at present, little is known about the putative SPS and SPP enzyme complex *in vivo.* Here, the possibility of protein interaction between *Arabidopsis thaliana* SPS and SPP was investigated to understand sucrose metabolism, as well as to provide evidence of an association between SPS and SPP. Additionally, the effects that overexpression of an *Arabidopsis SPS–SPP* fusion construct has on growth and sucrose metabolism in plants were examined.

## Materials and methods

### Plant material

*Arabidopsis thaliana* seeds (ecotype Columbia) and two T-DNA insertion mutants, *Atsps* ^*–*^ (CS85693) and *Atspp* ^*–*^ (CS859155), were surface-sterilized by first washing with 70% ethanol for 2min, followed by sodium hypochlorite for 30min, and finally with sterile distilled water. Sterilized seeds were then imbibed at 4 °C for 3 d in the dark, and then grown on Murashige and Skoog (MS) nutrient agar media containing 2% sucrose in a growth chamber (16/8h light/dark) at 23 °C. Seedlings were transferred to soil ~7 d post-germination, and grown in a growth chamber at 23 °C under a long-day light cycle (16h light/8h dark). When the plants were fully mature (yellowed siliques), they were harvested for cell wall analysis.

*Populus alba × P. grandidentata* (genotype P39) leaf discs were harvested from 4-week-old tissue culture-grown plants using a cork borer. Twenty plates containing 25 leaf discs (7mm^2^) per genotype were co-cultivated with 30ml of bacterial culture in 50ml Falcon tubes for 30min at 28 °C in a rotating shaker (100rpm). Following co-cultivation, the explants were blotted dry on sterile filter paper and placed abaxially on WPM culture medium containing 0.1 μM 1-naphthylacetic acid (NAA), 0.1 μM benzyladenine (BA), and 0.1 μM thidiazuron (TDZ). The plates were cultured in the dark for 2 d at room temperature. On the third day, residual *Agrobacterium* was eliminated by transferring the leaf discs to WPM medium containing 250mg l^–1^ cefotoxine and 500mg l^–1^ carbenicillin. All plates were kept in the dark for an additional 2 d. Explants were then transferred to WPM selection medium containing 250mg l^–1^ cefotoxine, 500mg l^–1^, carbenicillin, and 25mg l^–1^ hygromycin. After emergence, only one shoot per leaf disc was excised and placed on WPM selection medium. After 6 weeks growth, explants were transferred to fresh medium with the same composition, and permitted to develop. Plants were confirmed as transgenic by genomic DNA screening, and those identified as positive were then subcultured and multiplied on antibiotic-free WPM medium.

Transgenic trees were multiplied in WPM medium until ~8–10 plants of each transgenic event were of similar size, along with the appropriate control, non-transformed trees. The trees were then moved to 2 gallon pots containing perennial soil (50% peat, 25% fine bark, and 25% pumice; pH 6.0), where they were maintained on flood tables with supplemental lighting (16h days with 300 μmol s^–1^ m^–2^ supplemental lighting) and watered daily with fertilized water in the UBC greenhouse, Vancouver, BC. After 6 months greenhouse growth, tree height from the root collar to the apex of the tree was recorded, while stem diameter was measured using digital calipers at 10cm above the root collar (soil level). Transgenic lines and corresponding wild-type trees were harvested after 6 months of growth, and tissues were kept at –80 °C until evaluated.

### Yeast two-hybrid assay

The full-length cDNAs of *AtSPS5b* (At5g20280) and *AtSPP* (At2g35840) were amplified from whole plants of 2-week-old *A. thaliana*, and independently cloned into the gateway entry clone pENTR/D-TOPO (Invitrogen). The full-length cDNAs were amplified by using the following primer sets for *AtSPS* and *AtSPP*, respectively: forward SPSGWa1 (5’-CACCTACGTAATGGCCGGGAACGAT-3’) and reverse SPSGWb2 (5’-TCAGTCCTTGAGAAGCTCTAATTTC-3’); and forward SPPGWa1 (5’-CACCCCTAGGATGGAGCGTCTAACA TCT-3’) and reverse SPPGWb2 (5’- GCGGCCGCTCAGATGATC CAGATGCTAT-3’). Both cDNAs were transferred to pDEST 32, facilitating fusion to the GAL4 DNA-binding domain or transferred to pDEST 22 for fusion to the GAL4 activation domain. The sequence was confirmed for the paired constructs and introduced into *Saccharomyces cerevisiae* strain MaV203. As a positive control, the yeast two-hybrid combination consisting of transcript factor TT8 and MYB75 was used (a gift from Dr Brian Ellis; Bhargava *et al.*, 2010). Transformants consisting of pDEST32SPS coupled with pDEST22, and empty vector pDEST32 coupled with pDEST22 were employed as negative activation controls. Transformants were selected on SC/-Leu/-Trp (synthetic defined plate deficient for both Leu and Trp) plates. Positive interactions were determined by the ability of the transformed yeast cells to grow on SC-Leu-Trp-Ura, SC-Leu-Trp-His+10mM 3AT (3-aminotriazole) and SC-Leu-Trp+0.2% 5FOA (5-fluoroorotic acid) media. An X-gal colorimetric assay was used for determination of β-galactosidase activity. All media used in these studies were prepared according to ProQuest Two-Hybrid system protocols, and performed according to the manufacturer’s instructions (Invitrogen).

### Bioluminescence resonance energy transfer (BRET) construct development and assay

Constructs were made using Gateway cloning technology (Invitrogen). The N-terminal tagged human codonized *Renilla* luciferase destination BRET vector (35S_P_::hRLUC–attR, AY995142) and *AtSPS*, the clone generated in the yeast-two hybrid assay, were used to generate the 35S_P_::hRLUC–AtSPS vector. The 35S_P_::AtSPS–hRLUC construct was created using an *AtSPS* clone that was amplified by the primer combination, SPSGWb1 (5’-CCTAGGGTCCTTGAGAAGCTCTAAT-3’) and SPSGWa1 (5’-CACCTACGTAATGGCCGGGAACGAT-3’), and then transferred into the C-terminally tagged luciferase destination vector (35S_P_::attR–hRLUC, AY995143). The 35S_P_::AtSPP–YFP (yellow fluorescent protein) vector was made using the recombination of *AtSPP* (At2g35840) that was amplified using SPPGWa1 (5’-CACCCCTAGGATGGAGCGTCTAACATCT-3’) and SPPGWb1 (5’-GATGATCCAGATGCTATCATCCTTCACC-3’) into the C-terminally tagged YFP structure (35S_P_::attR–YFP, AY995145). A combination of the 35S_P_::AtSPP–YFP vector with either the 35S_P_::hRLUC–AtSPS or the 35S_P_::AtSPS–hRLUC vector was co-transformed into both the *Atspp* ^*–*^ and *Atsps* ^*–*^ mutant backgrounds using the floral dip method (Clough and Bent, 1998). A 35S_P_::RLUC vector and a 35S_P_::RLUC–YFP fusion vector (gifts from Dr A.G. von Arnim) were used as negative and positive controls, respectively. After YFP expression was confirmed in all four double transgenic lines, BRET signals were visualized in the root tissue of 8- to 9-day-old light-grown transgenic seedlings in the presence of 1 μM coelenterazine. Each sample was distributed into an individual well of a 96-well Optiplate (PerkinElmer Life Sciences) and emissions were measured using a Wallac Victor 3V plate reader (PerkinElmer Life Sciences). For BRET measurement, luciferase emission was measured through a 370–450nm filter and the resulting YFP emission was measured through a 500–530nm filter. Calculation of BRET was according to Subramanian *et al.* (2004). Readings from 4–5 plants from independent double transgenic lines were averaged and background subtracted using a reading from untransformed tissue.

### AtSPP–SPS fusion construct development and transformation

The *AtSPP* gene that was previously cloned was transformed into the pENTR/D-TOPO vector (Invitrogen) with forward primer SPPa1 (5’-CACCCCTAGGATGGAGCGTCTAACAT-3’) and reverse primer SPPb1 (5’-GATGATCCAGCTGCTATCATCCTTCA-3’) in order to include an N-terminal *Not*I restriction site and a C-terminal *Not*I restriction site before the stop codon. Similarly, the *AtSPS* gene that was previously cloned was transformed into the ZeroBlunt/TOPO vector (Invitrogen) with the forward primer SPSa3 (5’-CTCGAGACAAAATGGCCGGGAACGA-3’) and reverse primer SPSb3 (5’-GCGGCCGCAGCGTCCTTGAGAAGCT-3’) in order to include *Not*I sites. The *AtSPP* and *AtSPS* genes were then excised from their respective vectors and ligated together and placed back into the pENTR/D-TOPO vector for verification of correct orientation by sequencing. Using Gateway technology, the *AtSPP–AtSPS* fusion was then recombined into the 35S_P_::attR–YFP (AY995145) vector in order to create the 35S_P_::SPP–SPS–YFP vector. 35S_P_::SPP–SPS–YFP was then transferred to *A. tumefaciens* strain EHA105 by the freeze–thaw method. Transformation of hybrid poplar plant tissue was achieved using a standard leaf disk inoculation technique, as described above.

### Fluorescent microscopy

YFP expression of transgenic lines was imaged in 7-day-old seedlings for *Arabidopsis* and hybrid poplar. Images were collected on a fluorescence microscope (Zeiss Axiovert 200M, Germany) with a CCD camera (Hamamatsu Orca, USA). Each image shown represents a single focal plane. Images were acquired using Zeiss software, and were imported into Image J software (W. Rasband, National Institutes of Health, Besthesda, MD, USA) for cropping, contrast adjustment, and assembly.

### Bimolecular fluorescence complementation (BiFC) in transiently transformed tobacco leaf cells

BiFC expression vectors were created using Gateway vectors pCL112 and pCL113 (Invitrogen), which carry N-terminal (YN) and C-terminal (YC) fragments of YFP, respectively. *Atsps* (At5g20280) was inserted into pCL112 to create a YN-tagged *AtSPS* (YN-*SPS*), while *AtSPP1* (At1g51420), *AtSPP2* (At2g35840), *AtSPP3*(3a) (At3g54270), and *AtSPP4*(3b) (At3g52340) were inserted in pCL113 to create four independent YC-tagged *AtSPP* vectors (YC-*SPPx*). Tobacco leaves were transiently transformed with the YN-tagged *AtSPS* vector and one of the four YN-tagged *AtSPP* vectors to create four lines denoted as pCL112AtSPS–pCL113AtSPP*x* (*SPS–SPPx*). The positive control line was created using a 35S_P_::YFP vector, while negative control lines pCL112EM–pCL113EM (EM–EM), pCL112EM–pCL113AtSPP*x* (EM–*SPPx*), and pCL112AtSPS–pCL113EM (*SPS*–EM) were created using empty vector combinations. All transformations were conducted according to Sparkes *et al.* (2006). Briefly, leaves of 5-week-old tobacco (*Nicotiana tabacum*) were transformed by infiltration with *A. tumefaciens* carrying the expression vectors. YFP fluorescence was detected 48h and 65h post-infiltration on a Leica DMI6000 inverted microscope with a Quorum Wave FX system, which has a modified Yokogawa CSU-10 spinning disk scan head (Yokogawa Electric Corporation). YFP was excited with a 491nm laser and emissions were passed through a 528/38 band filter (Chroma Technology). Images were acquired using a Hamamatsu 9100–13 EMCCD camera (Hamamatsu) controlled by Volocity software (Improvision).

### Quantitative real-time PCR (RT-PCR)

Tissue was ground in liquid nitrogen using a mortar and pestle, and extracted with TRIzol^®^ reagent (Invitrogen) according to the manufacturer’s instructions. cDNA was synthesized using the Superscript™ II first strand synthesis system (Invitrogen) in a 20 μl reaction containing 1 μg of DNase I-treated total RNA with 0.5 μg of dT_16_ oligonucleotides. An identical reaction without the reverse transcriptase was performed to confirm the absence of genomic DNA. Transcript abundance was quantified with Brilliant^®^ SYBR^®^ Green QPCR Master Mix (Stratagene, La Jolla, CA, USA) on an Mx3000P™ PCR system (Stratagene) employing the forward and reverse primers: ASPS-F3 (5’-CCACAGTGGCAAAGTGATGATGGC-3’) and ASPS-R4 (5’-TCTGACCTCTCCAGTGATCCC-3’), ASPP-F3 (5’-GTGGTGTGTTTGTCCATCCATGTG-3’), and ASPP-R3 (5’-ACTTGATCTGCCCAAACGCGAAAC-3’). The *Arabidopsis ubiquitin5* gene (*AtUBQ5*; At3g62250) served as a housekeeping control, and was amplified using the following forward and reverse primers: AtUBQF (5’-ACGCCAAGCCGAAGATCAAGCA-3’) and AtUBQR (5’-AAATGACTCGCCATGAAAGTCCCAG-3’). The thermocycler conditions for all real-time analyses were: 95 °C for 10min, followed by 40 cycles of 95 °C for 30 s, 55 °C for 1min, and 72 °C for 30 s. Data were analysed using Mx3000P™ Real-Time PCR system software (Stratagene). Transcript abundance of *Atsps* and *Atspp* was normalized to *ubq5* by subtracting the Ct value of *ubq5* from the Ct value of the specific gene, where ∆Ct=Ct_*AtSPS*_ or Ct_*AtSPP*_–Ct_*AtUBQ5*_. Transcript abundance of the *Atsps* gene in wild-type and transgenic plants was obtained from the equation (1+E)^–∆Ct^, where E is the PCR efficiency, according to Ramakers *et al.* (2003), which is derived from the log slope of the fluorescence versus cycle number in the exponential phase of each individual amplification plot, using the equation (1+E)=10^slope^.

### Soluble sugar and starch analysis

Leaf tissues were harvested and immediately frozen in liquid nitrogen, ground with a mortar and pestle, and then freeze-dried for 48h. A 20–50mg aliquot of tissue was incubated for 24h at –20 °C with 4ml of methanol:chloroform:water (12:5:3). The samples were centrifuged for 10min at 6000rpm and 4 °C, and the supernatant was collected. The pellet was washed with 8ml of methanol:chloroform:water (12:5:3), centrifuged for an additional 10min at 6000rpm and 4 °C, and the supernatants were pooled. An aliquot of 5ml of distilled water was then added to the pooled supernatants and phases were partitioned, at which point 1ml of the resulting aqueous phase containing the soluble sugars was dried in a speedvac at 40 °C. The pellet was resuspended in 1ml of nanopure water and filtered through a 4mm nylon filter (0.45 μm). The soluble sucrose concentrations were quantified by anion-exchange high-performance liquid chromatography (HPLC; Dionex, Sunnyvale, CA, USA) on a DX-600 equipped with a CarboPac™ PA1 column and an electrochemical detector. Sugars were eluted with water at room temperature at a flow rate of 1ml min^–1^. Each concentration was determined using regression equations from calibration curves that were derived from external standards. For the hybrid poplar trees, starch content was determined by adding 3ml of 4% H_2_SO_4_ to ~60mg of the solid tissue remaining after the soluble sugar extraction and then autoclaving for 4min at 121 °C. Glucose content was then determined by HPLC, as described below, for the structural carbohydrate analyses.

### Structural carbohydrate, lignin, and starch analysis

Tissue samples harvested from ~5cm of the base of 6-month-old greenhouse-grown hybrid poplar trees were ground in a Wiley mill to pass a 0.4mm screen (40 mesh) and Soxhlet extracted overnight in hot acetone to remove extractives. The extractive-free material was used for all further analyses.

The lignin and carbohydrate content was determined with a modified Klason (Coleman *et al.*, 2008), in which extracted ground stem tissue (0.2g) was treated with 3ml of 72% H_2_SO_4_ and stirred every 10min for 2h. Samples were then diluted with 112ml of deionized water and autoclaved for 1h at 121 °C. The acid-insoluble lignin fraction was determined gravimetrically by filtration through a pre-weighed medium coarseness sintered-glass crucible, while the acid-soluble lignin component was determined spectrophotometrically by absorbance at 205nm. Carbohydrate content was determined by using anion-exchange HPLC (Dx-600; Dionex) equipped with an ion-exchange PA1 (Dionex) column, a pulsed amperometric detector with a gold electrode, and a Dionex AS100 autosampler (Dionex).

### Wood density

Wood density was determined using bark-to-bark segments isolated from 15cm above the root collar and precision cut to 1.67mm thick specimens with a precision custom-built twin-blade pneumatic saw. After extraction with hot acetone in a Soxhlet apparatus overnight, the wood sections were acclimated to 7% moisture content prior to density determination. The samples were then scanned by X-ray densitometry at a resolution of 0.254mm (QTRS-01X; Quintek Measurement Systems Inc., Knoxville, TN, USA).

## Results

### Yeast two-hybrid assay

**Fig. 1.**
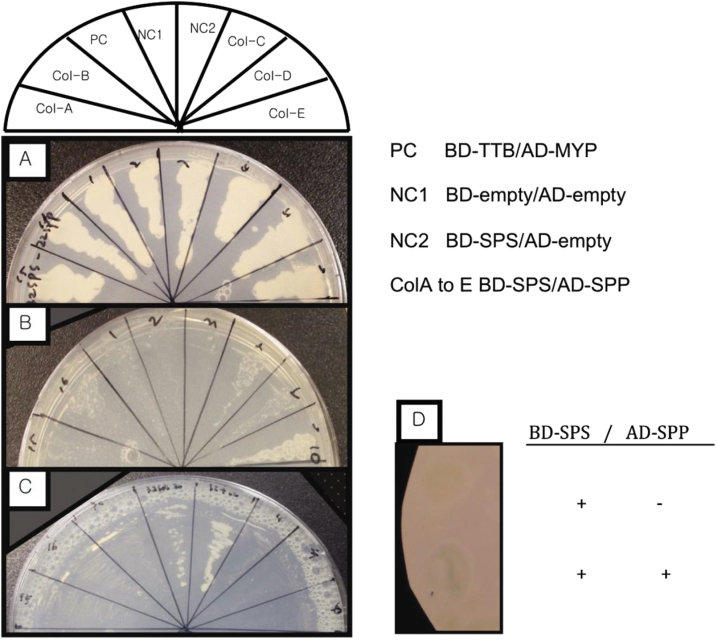
Protein interaction of *Arabidopsis* AtSPS (At5g20280) and AtSPP (At2g35750) using a yeast two-hybrid assay. (A) SC/-Leu/-Trp plate confirming the introduction of both plasmids into the cells. (B) Prototrophic growth of the yeast two-hybrid strain MaV203 on media depleted in histidine+10mM 3AT indicates positive interactions. (C) Growth inhibition of cells containing interacting proteins results from induction of URA3 reporter genes on medium containing 0.2% 5-fluoroorotic acid (5FOA). (D) Induction of the *lacZ* gene with X-gal resulted in a blue colour, indicating that a positive protein interaction between AtSPS and AtSPP was produced.

In an attempt to assess the putative protein–protein interaction between AtSPS and AtSPP, the full-length *AtSPS* cDNA fused in-frame with the DNA-binding domain of GAL4 and the *AtSPP* fused in-frame with the GAL4 activation domain were used for co-transformation in a direct yeast two-hybrid assay system. Transformed colonies were first selected on medium lacking leucine and tryptophan, confirming the introduction of both plasmids into cells (Fig. 1A), while positive interactions were detected by selection on plates lacking the auxotrophic markers histidine or uracil. These cells were then grown on selective medium lacking histidine, which included 10mM 3AT as a HIS3 inhibitor (Fig. 1B). In order to monitor the putative positive interaction further, the cells were grown on media with 0.2% 5FOA. The positive yeast cell lines, pDEST™ 32/*SPS* and pDEST™ 22/*SPP*, produce the two-hybrid-dependent induction of URA3, which results in the conversion of 5FOA to 5-fluorouracil, which is toxic. Hence, the AtSPS and AtSPP proteins in the cells containing both plasmids must be interacting, as indicated by their inability to grow on medium containing 5FOA (Fig. 1C). Furthermore, induction of the *lacZ* gene with X-gal (5-bromo-4-chloro-3-indolyl-β-d-galactopyranoside) using the colorimetric assays for β-galactosidase resulted in a blue colour, indicating that a positive protein interaction between AtSPS and AtSPP was produced. The combination of pDEST32-*SPS* and the empty vector pDEST22 served as a negative interaction control and, as expected, showed no interaction (Fig. 1D). The combination of empty vectors pDEST32 and pDEST22 served as additional negative controls on each selective medium.

### BRET assay *in planta*

**Fig. 2.**
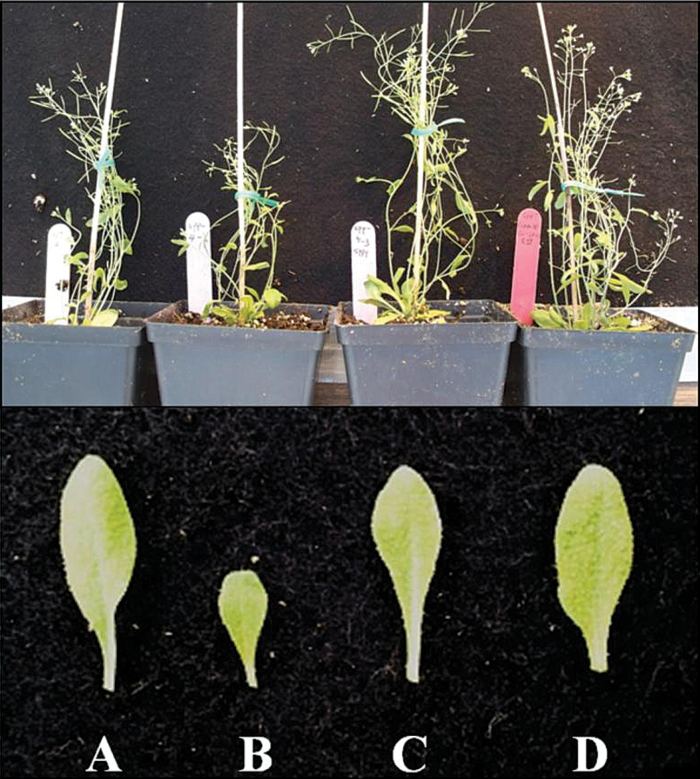
Complementation of the *Atspp* ^*–*^ mutant line indicates functionality of each BRET construct. (A) Wild type, (B) *Atspp* ^*–*^ mutant, (C) SPP^Y^ in the *Atspp* ^*–*^ mutant, and (D) ^*R*^ *SPS-SPP* ^*Y*^ in the *Atspp* ^*–*^ mutant.

**Fig. 3.**
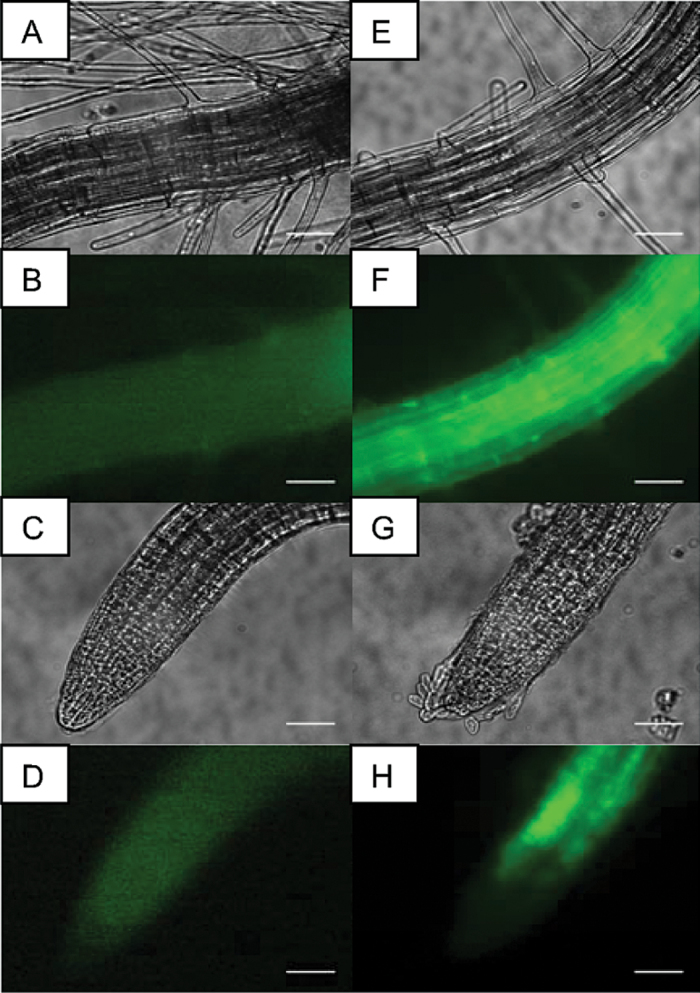
YFP fluorescence in root tissue from 7-day-old transgenic plants expressing *SPS* ^*R*^ *SPP* ^*Y*^ provides confirmation that the constructs were functional as well as proving proof that YFP fusion proteins can be expressed stably in *Arabidopsis* seedlings. (A–D) Control *Atsps* ^*–*^ mutant plant, (E–H) a double transgenic plant expressing both SPS^R^ and SPP^Y^ in hairy root and root tip tissue. Scale bar=50 μm.

To support the yeast two-hybrid results, the capacity of AtSPS and AtSPP to interact *in planta* was studied using the BRET technique. The functionality of each BRET construct described previously was confirmed by complementation of the dwarf phenotype of the respective mutants (Figs 2, 8). Four double transgenic lines were then created by co-transforming either the *Atsps* ^*–*^ mutant or the *Atspp* ^*–*^ mutant with either the 35S_P_::AtSPS-hRLUC or 35S_P_::hRLUC::AtSPS construct and the 35S_P_::AtSPP–YFP construct, respectively. Before the BRET assay was conducted, the double transgenic lines were screened by genomic DNA PCR and subsequently YFP expression was examined by fluorescence microscopy. YFP fluorescent signals were observed clearly in most of the root tissue, excluding the root tip region in the transgenic plants, providing further confirmation that the constructs were functional, as well as providing proof that YFP fusion proteins can be expressed stably in *Arabidopsis* seedlings (Fig. 3).

**Fig. 4.**
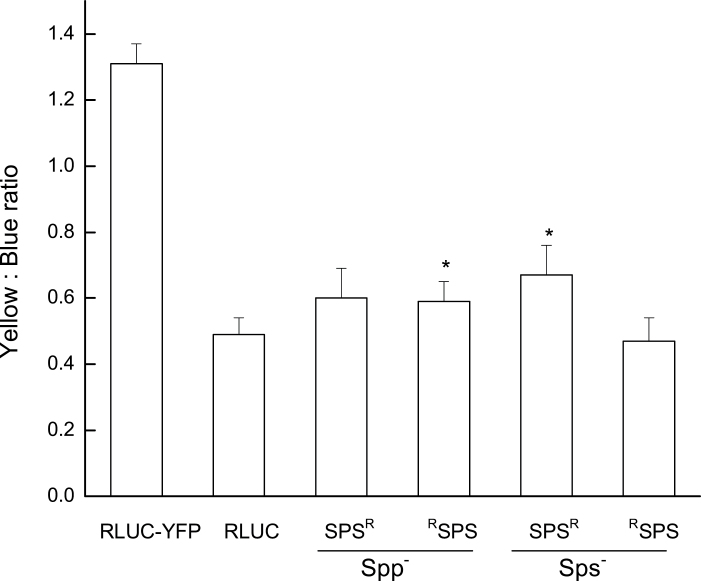
BRET signal ratio in *Arabidopsis* hypocotyls from seedlings 7 d after germination using 1 μM coelenterazine at 22 °C indicates a significant (*P*<0.05) difference in the BRET ratio of ^R^SPS in the *Atspp* ^*–*^ mutant background and SPS^R^ in the *Atsps* ^*–*^ mutant background, when compared with the RLUC negative control. P_35S_::hRLUC·YFP and P_35S_::hRLUC structures were transformed for a positive control and negative control, respectively.

Following confirmation of YFP expression, a BRET assay was conducted on the double transgenic lines. In a BRET experiment, the extent of energy transfer from RLUC to YFP is measured via a luminometer by filtering the signal luminescence through blue and yellow filters, respectively, and calculating the ratio between yellow and blue signals. Here, the yellow/blue (Y/B) luminescence ratios of 10-day-old light-grown double transgenic seedlings, as well as the negative and positive controls, were calculated. The resultant BRET ratio of the C-tagged RLUC AtSPS in the *Atsps* ^*–*^ mutant background and the N-tagged RLUC AtSPS in the *Atspp* ^*–*^ mutant background was significantly higher than that of the RLUC fusion expressed alone as a negative control in the mutant backgrounds (Fig. 4). There was a slight signal discrepancy, of ~0.2, when comparing the interaction in the different mutant backgrounds, with slightly stronger signals in the *Atspp* ^*–*^ mutant background. According to Subramanian *et al.* (2006), a BRET signal is considered positive when the yellow to blue ratio is >0.04 compared with the RLUC fusion expressed alone as a negative control. Here, the majority (70%) of 50 pairings of gene products tested showed a ratio >0.04, with some as high as 0.3. These findings suggest a positive protein–protein interaction with these constructs. However, a significant difference in the BRET ratio was not seen in N-tagged RLUC *AtSPS* in the *Atsps* ^*–*^ mutant background and the C-tagged RLUC *AtSPS* in the *Atspp* ^*–*^ mutant background. While the exact reason for this is unknown, it has been suggested that an unfavourable orientation of the emission dipoles of RLUC and YFP or an excessive distance due to their spatial separation by the larger fused proteins will abolish the BRET signal (Subramanian *et al*., 2004, 2006). These data taken together with the protein–protein interaction results observed in the yeast two-hybrid assays suggest that there is an active interaction between AtSPS and AtSPP.

### BiFC in transiently transformed tobacco epidermal cells

**Fig. 5.**
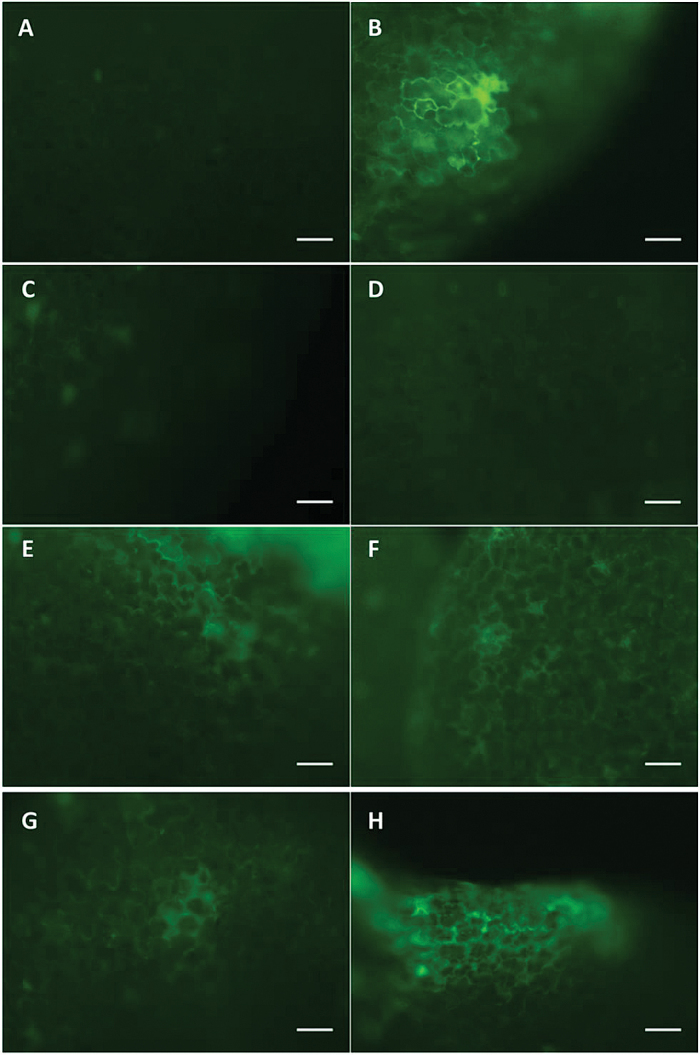
The YFP signal in 5-week-old *Nicotiana tabacum* cells transiently transformed with BiFC constructs shows interaction between AtSPS and AtSPP proteins. (A) EM–EM negative control, (B) 35S_P_ –YFP positive control, (C) SPS–EM negative control, (D) EM–SPP1 negative control, (E) SPS–SPP1, (F), SPS–SPP2, (G) SPS–SPP3, and (H) SPS–SPP4. Scale bar=20 μm.

While the previous yeast two-hybrid and BRET results strongly suggest an interaction between AtSPS and AtSPP, additional evidence was sought by using a BiFC assay to detect AtSPP–AtSPS interactions visually *in planta*. In BiFC, two proteins that are believed to interact are fused to different non-fluorescent fragments of a fluorescent protein (i.e. YFP) and expressed in live cells. If the proteins of interest do in fact interact, they will facilitate the re-formation of the fluorescent protein enabling it to emit its fluorescent signal which can then be visualized using fluorescence microscopy (Kerppola, 2006). In this study, leaves from 5-week-old *N. tabacum* were transiently transformed with YN-*SPS* and either YC-*SPP1*, *2*, *3*, or *4*. After infiltration with the BiFC-tagged combinations of *AtSPS–AtSPPx*, leaf tissue was scanned for fluorescence as an indication of AtSPS–AtSPS interactions. 35S_P_::YFP was used as a positive control while EM–EM, EM–*SPPx*, and *SPS*–EM were used as negative controls. It was determined that the optimal time to detect the YFP signal was between 48h and 65h post-infiltration. YFP signal was clearly detected in all of the SPS–SPP lines, providing additional evidence for an interaction between these two proteins (Fig. 5).

### Relative transcript of *AtSPS* and *AtSPP* in transgenic *Arabidopsis* plants

**Fig. 6.**
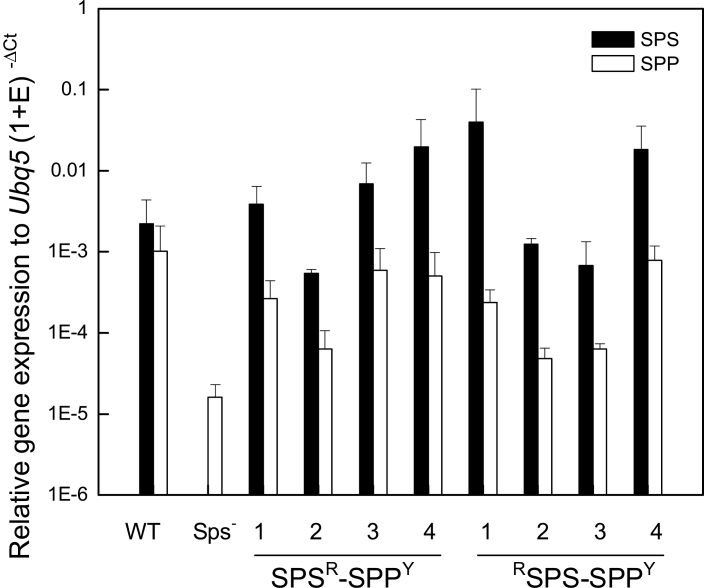
Real-time PCR analysis of *AtSPS* (At5g20280) and *AtSPP* (At2g35750) gene expression in rosette leaves of *Arabidopsis* indicates increased expression of both transgenes when compared with the *Atsps* ^*–*^ mutant. *AtSPS* (At5g20280) was tagged with hRLUC in N- and C-terminal orientations (SPS^R^ and ^R^SPS), and *AtSPP* (At2g35740) was tagged with YFP at the C-terminus (SPP^Y^). The *Arabidopsis ubiqutin5* gene was used as an internal control, and results are given as relative transgene transcript abundance.

Double transgenic *Arabidopsis* plants in the *Atsps* ^*–*^ mutant background were analysed by quantitative RT-PCR to assess the transcript abundance of each cisgene. *AtSPS* and *AtSPP* transcripts was quantified in rosette leaf tissue for all transformed lines and normalized to *AtUBQ5* abundance (Fig. 6). No *AtSPS* transcript was observed in the *Atsps* ^*–*^ mutant as expected, and therefore all transcripts measured were due to the expression of the *AtSPS* cisgene. Interestingly, the *AtSPP* transcript level in the *Atsps* ^*–*^ mutant plant was also reduced compared with wild-type plants. In all cisgenics, the *AtSPS* transcript abundance was apparent, and the transcript abundance of *AtSPP* was similar among all lines, which was slightly higher than in the *Atsps* ^*–*^ mutant plants.

### AtSPP–SPS in hybrid poplar

**Fig. 7.**
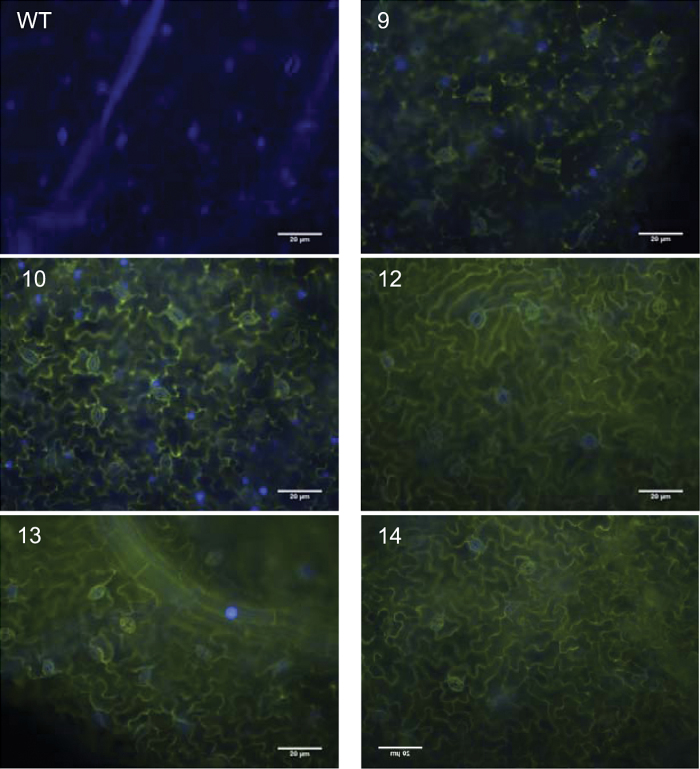
YFP signal in *AtSPP-SPS-YFP* poplar plants indicates functionality of the 35S_P_::SPP–SPS–YFP vector.

In order to confirm the functionality of the 35S_P_::*SPP-SPS*-*YFP* vector, the presence of YFP fluorescence was confirmed using microscopy (Fig. 7). A strong YFP signal in all of the transgenic hybrid poplar lines clearly indicates that the foreign construct is being expressed and that the proteins are being produced.

### Plant growth

**Fig. 8.**
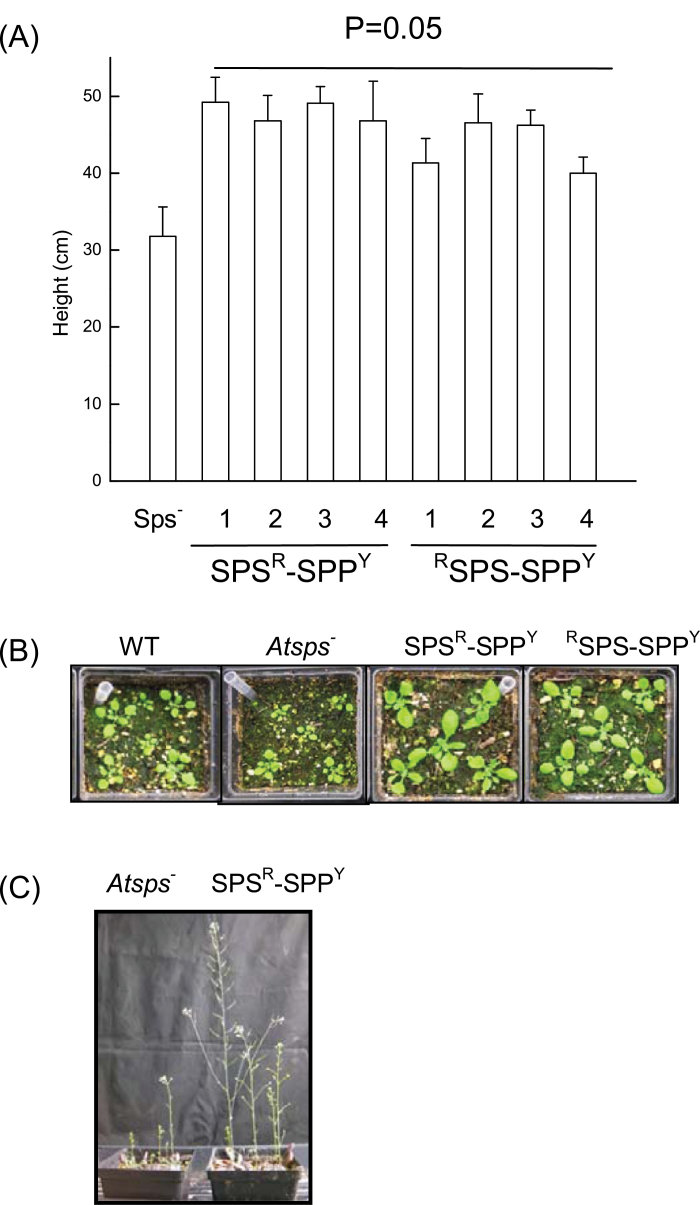
Growth comparison of *Atsps* ^–^ *Arabidopsis* plants expressing *AtSPS* and *AtSPP* simultaneously indicates their ability to outperform the *Atsps* ^*–*^ mutant, as well as the wild-type plants. (A) Height measurements of 2-month-old SPS^R^:SPP^Y^ and ^R^SPS:SPP^Y^ *Arabidopsis* plants compared with the *Atsps* ^*–*^ mutant plant. Error bars indicate the standard deviation. The line denotes significance at *P*=0.05. (B) Transgenic *Arabidopsis* seedlings. (C) Complementation of the *Atsps* ^–^ mutant line with SPS^R^–SPP^Y^.

**Fig. 9.**
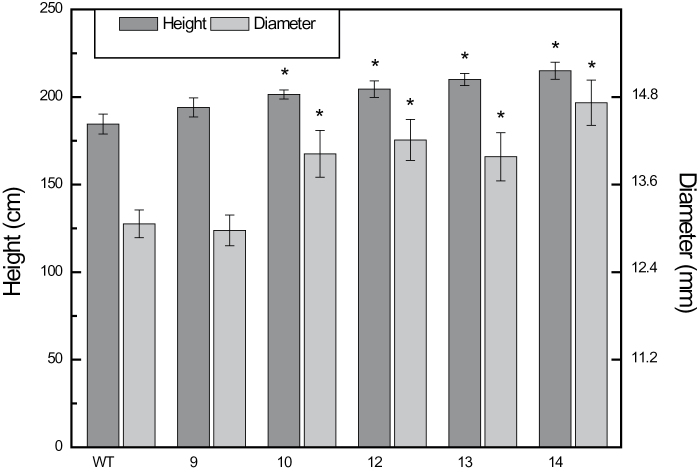
Height and stem diameter measurements of hybrid poplar trees after 4 months of growth in the greenhouse indicates increased growth in the AtSPS–SPP fusion lines. Error bars are the standard error of the mean, *n* ≥6. * indicates *P*>0.05 using a Students *t*-test.

Increasing the activity of AtSPS and AtSPP affected plant growth rates in both *Arabidopsis* and hybrid poplar. In general, the height of the *Arabidopsis* cisgenic lines expressing *AtSPS* and *AtSPP* under the regulation of the 35S promoter in the *Atsps* ^*–*^ mutant background outperformed the wild type, as well as the *Atsps* ^*–*^ mutant lines, grown under similar conditions (Fig. 8). *Arabidopsis* plants transformed with two fused BRET constructs had >50% higher growth than the *Atsps* ^*–*^ mutant plants or the wild type (Fig. 8A). Moreover, the lines that were shown to have significant increases in the BRET ratio (>0.03) displayed substantially faster growth rates than the control (wild-type or *Atsps* ^*–*^) plants (Fig. 8C). In hybrid poplar, the expression of the 35S::*AtSPS-SPP* fusion construct significantly increased both height and stem diameter in four of the five transgenic lines studied (Fig. 9). In contrast, expression of either AtSPP (data not shown) or AtSPS (Park *et al.*, 2009) on their own in hybrid poplar did not manifest in increase in growth. In addition to the increased height and diameter growth, the wood density of the xylem was increased in all transgenic lines, as determined by X-ray densitometry (Table 1). Effectively, the combined growth increase with the enhanced cell wall density significantly increases the total biomass of the transgenic poplar trees over the wild-type controls growing concurrently.

**Table 1.** *Wood density of stem segments of* AtSPS–SPP *transgenic and wild-type poplar trees*

Line	Wood density (kg m^–3^)
WT	309.4±12.9
9	337.1±22.1
10	**343.9±14.8**
12	**345.6±10.4**
13	**351.2±8.8**
14	339.9±17.9

Means (± SE) were calculated from five plants per line.

Bold denotes a significant difference from the control at α=0.05.

### Soluble sugar and starch analysis

**Fig. 10.**
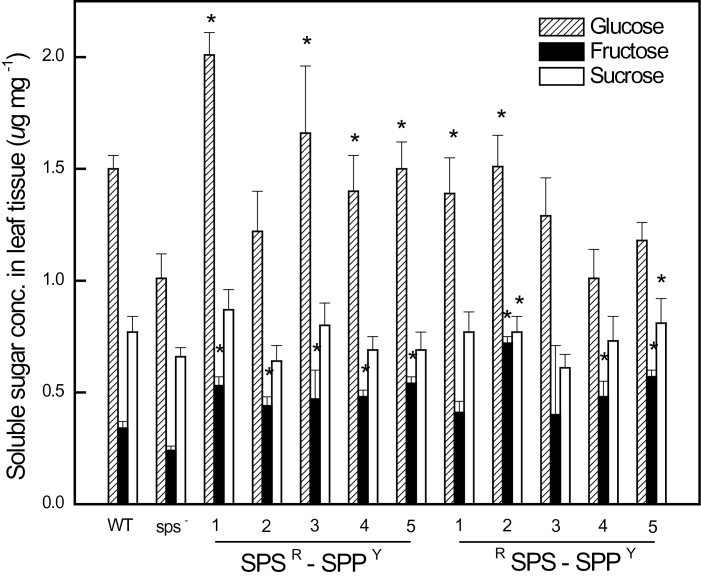
Comparison of soluble sugar concentrations in *Atsps* ^*–*^ *Arabidopsis* rosette leaf tissue expressing *AtSPS* and *AtSPP* simultaneously indicates an increase in these sugars when compared with the *Atsps* ^*–*^ mutant, as well as the wild type. Data are means (± SD) of five independent plants per line. * denotes significance at *P*=0.05 compared with *Atsps* ^*–*^ mutant plants.

In order to analyse the influence of increased AtSPS and AtSPP activity on sucrose metabolism in the cisgenic plants, soluble sugars from the *Atsps* ^*–*^ mutants, wild-type, and cisgenic plants were extracted and analysed. The concentration of leaf sucrose in all the SPS–LRUC and SPP–YFP cisgenic lines, which showed an *in planta* BRET signal, was slightly elevated compared with wild-type and mutant plants while, glucose and fructose pools were dramatically increased. The constructs that did not show significant increases in the BRET ratio also had increased soluble sugar content; however, the increase was smaller (Fig. 10). Similarly, the hybrid poplars overexpressing the *AtSPP–SPS* fusion construct had an increase in the fructose and glucose pools, especially in the source leaves (Table 2). The starch content in both the source and sink leaves of the *AtSPP–SPS* transgenic hybrid poplar was also reduced (Table 3).

**Table 2.** *Comparison of soluble sugar concentrations in transgenic* AtSPS–SPP *and wild-type hybrid poplar source and sink leaves*

Line	Soluble sugar extracted (mg) mg^–1^ tissue (%)					
	Glucose		Fructose		Sucrose	
	Source	Sink	Source	Sink	Source	Sink
WT	0.95 (0.09)	0.36 (0.05)	0.66 (0.11)	0.34 (0.06)	4.03 (0.86)	0.79 (0.036)
9	**1.59 (0.37**)	0.54 (0.08)	**1.59 (0.46**)	0.55 (0.15)	4.99 (0.47)	0.96 (0.23)
10	**1.36 (0.13**)	0.38 (0.03)	**1.11 (0.12**)	0.45 (0.03)	5.02 (0.24)	1.16 (0.32)
12	1.06 (0.20)	0.48 (0.14)	0.80 (0.12)	0.40 (0.08)	4.84 (0.06)	1.64 (0.68)
13	**1.34 (0.08**)	0.48 (0.09)	0.81 (0.07)	0.49 (0.07)	4.99 (0.15)	0.78 (0.43)
14	**1.37 (0.12**)	0.32 (0.10)	**1.74 (0.04**)	0.33 (0.09)	4.60 (0.03)	0.72 (0.29)

Data are means (± SEM) of five biological replicates

Bold denotes a significant difference at *P*=0.05 using a Student *t*-test.

**Table 3.** *Starch content of 6-month-old transgenic* AtSPS–SPP *and wild-type hybrid poplar source and sink leaves*

Line	Starch (% of dry mass)	
	Sink	Source
WT	1.70 (0.27)	5.50 (1.01)
9	**1.36 (0.18**)	4.82 (0.75)
10	1.29 (0.46)	**2.92 (0.11**)
12	**0.95 (0.32**)	4.74 (1.58)
13	**1.38 (0.11**)	5.02 (0.84)
14	**1.02 (0.09**)	4.60 (0.53)

The SEM is given in parentheses, *n*=5 biological replicates.

Bold denotes a significant difference at *P*=0.05 using a Student *t*-test.

### Structural carbohydrate and lignin analysis

Structural carbohydrates and lignin composition were determined from extractive-free dried stem tissue of 6-month-old hybrid poplar trees to investigate the effects that the expression of the AtSPP–SPS fusion protein had on carbohydrate-derived polymer synthesis (Table 4). Although none of the *AtSPP–SPS* transgenic lines shows a striking difference in either carbohydrate composition or lignin content, significant differences in total lignin content were observed, with two lines (10 and 13) showing a statistically significant increase and the other three transgenic lines also having increasing concentrations. Similarly, while the hemicellulose and pectic-derived structural carbohydrates were unaltered, the total cell wall glucose content was elevated (Table 4).

**Table 4.** *Structural cell wall carbohydrates and total lignin content of 6-month-old* AtSPS–SPP *and wild-type hybrid poplar*

Line	Carbohydrates (% of dry mass)						Lignin (% of dry mass)		
	Arabinose	Rhamnose	Galactose	Glucose	Xylose	Mannose	Insoluble	Soluble	Total
WT	0.35 (0.01)	0.24 (0.04)	1.23 (0.12)	44.75 (1.04)	17.05 (0.39)	1.97 (0.15)	18.69 (0.63)	3.81 (0.16)	22.50 (0.75)
9	0.36 (0.03)	0.27 (0.04)	1.13 (0.04)	46.20 (0.72)	17.37 (0.21)	2.02 (0.11)	19.84 (0.35)	3.83 (0.03)	23.68 (0.37)
10	0.33 (0.01)	0.28 (0.03)	1.05 (0.03)	45.97 (0.72)	17.62 (0.13)	1.83 (0.04)	**20.51 (0.31**)	**4.04 (0.17**)	**24.55 (0.22**)
12	0.35 (0.02)	0.26 (0.03)	1.14 (0.06)	45.26 (0.83)	17.49 (0.42)	2.09 (0.10)	19.51 (0.38)	3.89 (0.22)	23.40 (0.35)
13	0.34 (0.01)	0.25 (0.05)	1.08 (0.05)	46.41 (0.65)	17.69 (0.17)	2.00 (0.03)	**20.50 (0.25**)	**3.87 (0.24**)	**24.36 (0.31**)
14	0.32 (0.01)	0.26 (0.04)	1.13 (0.02)	**48.41 (0.29**)	17.16 (0.24)	1.99 (0.14)	**19.98 (0.34**)	3.85 (0.18)	23.84 (0.43)

The SEM is given in parentheses, *n*=5 biological replicates.

Values in bold have a *t*-test *P*-value of ≤0.05 relative to the wild type.

## Discussion

While previous studies have implied that an interaction between SPP and SPS proteins is not only beneficial for plant development, but also probable (Echeverria *et al.*, 1997; Elcock *et al.*, 1997; Piotrowski and Oecking, 1998; Toroser *et al.*, 1998), to date there has been no *in planta* evidence that this interaction exists. This study therefore was designed to thoroughly investigate the putative interaction between SPS and SPP, and assess the impact of misregulating both genes simultaneously on plant growth and development. The current results using the yeast two-hybrid assay indicated that SPS and SPP do in fact interact, and further evidence was provided using BRET and BiFC assays (Figs 1, 3, 4). Previous enzymatic studies have indicated that there is indeed a significant advantage in having two enzymes working in concert with one another to form a metabolic channel. The two-domain structure of SPS, an N-terminal glycosyltransferase and a C-terminal phophatase domain, has a remarkable resemblance to that of the enzymes required for the synthesis of the disaccharide trehalose. The biosynthesis of trehalose involves a two-step process catalysed by trehalose-6-phosphate synthase (TPS) and trehalose-6-phosphate phosphatase (TPP). Trehalose-6-phosphate (T6P) is formed by TPS and is then dephosphorylated to trehalose by TPP. Seo *et al.* (2000) demonstrated that an interaction between TPS and TPP, as well as the catalytic efficiency of the two fusion enzymes, was up to 4.0 times higher than that of a mixture of individual enzymes. A physical interaction of TSP and TPP would permit the low abundant T6P to channel from one enzyme to the other without the need to equilibrate with the bulk solution. This mechanism has been explained by the presence of an electrostatic channel on the surface of the proteins that connect the two active sites (Elcock *et al.*, 1997). Recently, T6P has been shown to have a profound effect on plant growth and development, acting as a signal of sugar availability and possibly the overall sucrose status (Yadav *et al.*, 2014). Similarly, a physical interaction between SPS and SPP could facilitate the channelling of Suc-6-P from one enzyme to the other without diffusing freely in solution. Furthermore, physical and kinetic evidence for an association between the two proteins suggested that the presence of SPP did not alter the affinity of SPS for its substrates, but aided in reversing the Pi inhibition at low Fru-6-P levels. Additional evidence from isotope dilution experiments suggests that Suc-6-P is indeed channelled between SPS and SPP (Salerno *et al.*, 1996; Echeverria *et al.*, 1997). A significant advantage to this arrangement would be that enzyme activity in the synthesis of sucrose would dramatically increase when compared with the enzyme activity of individual proteins.

In addition to providing evidence for the presence of an SPP–SPS protein interaction, it was possible to demonstrate that a concurrent increase in *AtSPS* and *AtSPP* expression in the *Atsps* ^*–*^ mutant background enabled the plants to outperform their wild-type controls, as well as the *Atsps* ^*–*^ mutant line, grown concurrently. Similarly, expression of an *AtSPP–SPS* chimera fusion construct in hybrid poplar also manifested in faster tree growth in both height and diameter. These findings suggest that changing a plant’s ability to synthesize sucrose by simultaneously overexpressing *SPS* and *SPP* is sufficient to trigger changes in the entire gene cascade to promote rapid plant growth and development. Häusler *et al*. (2000) suggested that increased growth and yield of plants with higher SPS activity may be the result of decreased energy costs associated with direct export of sucrose during the day rather than the synthesis of starch and its conversion back to sucrose for export at night. Consistent with the hypothesis of Häusler *et al*. (2000), here not only was a decrease in starch content observed in sink leaves of the *AtSPP–SPS* hybrid poplar trees, but lower starch contents were also observed in the source leaves. Alternatively, the increased soluble carbohydrate status caused by elevated SPS activity may signal the redistribution of resources to actively growing sink regions of the plant (Laporte *et al.*, 2001). While there was only a slight increase in sucrose content in the *AtSPS*- and *AtSPP*-overexpressing plants, fructose and glucose significantly increased in most lines when compared with the *Atsps* ^–^ mutant and wild-type plants. It appears that the action of the elevated SPS and SPP activity manifests in the synthesis of sucrose that is rapidly broken down to glucose and fructose by either invertase or sucrose synthase counterparts, and the overall level of sucrose maintains a homeostatic level. Concurrent with the evidence of a protein interaction between SPS and SPP, increased growth rates and altered soluble sugar contents in the lines overexpressing both *AtSPS* and *AtSPP* could be indicative of a further increase in the effectiveness of sucrose synthesis. Similarly, transformation experiments in tomato, tobacco, sugarcane, and *Arabidopsis* have suggested that when the activity of an SPS is elevated in transgenic plants, it alters *in planta* sucrose status, manifesting in enhanced growth (Worrell *et al.*, 1991; Micallef *et al.*, 1995; Laporte *et al.*, 1997, 2001; Sharkey *et al.*, 2004; Park *et al.*, 2008; Coleman *et al.*, 2010). It has also been shown that SPS activity strongly influences the partitioning of photosynthetic carbon in *Zea mays* leaves, and that the effect of SPS on growth was a consequence of a shift in partitioning of carbon among starch, sucrose, and ionic compounds, rather than due to an increase in net photosynthesis (Lunn and Hatch, 1997).

While it is fair to conclude that an interaction between SPS and SPP does exist in *Arabidopsis* and the overexpression of both genes leads to increased growth and soluble carbohydrates in both *Arabidopsis* and hybrid poplar, further work is needed to clarify the molecular basis of protein binding with respect to differences among SPS and SPP isoforms and crucial structural elements. It would also be important to test whether other proteins involved in the sucrose synthesis pathway, such as sucrose synthase, form multienzyme complexes with these interacting enzymes. Coleman *et al.* (2009) showed that an overexpression of sucrose synthase in transgenic poplar manifested in increased cellulose content, but without an increase in overall growth characteristics. Moreover, the transgenic sucrose synthase poplar had increased cell wall densities. Herein, it is shown that the *SPS–SPP* chimera could increase plant growth attributes, which also had positive affects on xylem density and cellulose composition. These findings support the possibility of a multienzyme complex regulating sucrose partitioning to cellulose biosynthesis. Importantly, these findings suggest that altering sucrose metabolism may be key to improving the biomass quantity and quality for bioenergy applications.
